# Emotion induction in young and old persons on watching movie segments: Facial expressions reflect subjective ratings

**DOI:** 10.1371/journal.pone.0253378

**Published:** 2021-06-18

**Authors:** Svenja Zempelin, Karolina Sejunaite, Claudia Lanza, Matthias W. Riepe

**Affiliations:** Department of Psychiatry and Psychotherapy II, Mental Health & Old Age Psychiatry, Ulm University, Ulm, Germany; Universidad Complutense Madrid, SPAIN

## Abstract

Film clips are established to induce or intensify mood states in young persons. Fewer studies address induction of mood states in old persons. Analysis of facial expression provides an opportunity to substantiate subjective mood states with a psychophysiological variable. We investigated healthy young (YA; n = 29; age 24.4 ± 2.3) and old (OA; n = 28; age 69.2 ± 7.4) participants. Subjects were exposed to film segments validated in young adults to induce four basic emotions (anger, disgust, happiness, sadness). We analyzed subjective mood states with a 7-step Likert scale and facial expressions with an automated system for analysis of facial expressions (FaceReader^™^ 7.0, Noldus Information Technology b.v.) for both the four target emotions as well as concomitant emotions. Mood expressivity was analysed with the Berkeley Expressivity Questionnaire (BEQ) and the Short Suggestibility Scale (SSS). Subjective mood intensified in all target emotions in the whole group and both YA and OA subgroups. Facial expressions of mood intensified in the whole group for all target emotions except sadness. Induction of happiness was associated with a decrease of sadness in both subjective and objective assessment. Induction of sadness was observed with subjective assessment and accompanied by a decrease of happiness in both subjective and objective assessment. Regression analysis demonstrated pre-exposure facial expressions and personality factors (BEQ, SSS) to be associated with the intensity of facial expression on mood induction. We conclude that mood induction is successful regardless of age. Analysis of facial expressions complement self-assessment of mood and may serve as a means of objectification of mood change. The concordance between self-assessment of mood change and facial expression is modulated by personality factors.

## Introduction

The concept of primary affects or basic emotions has been under consideration for a long time [1, 2]. One of the main characteristics of this concept is that basic emotions are distinct from one another and are accompanied by specific physiological states, facial features, or other physiological markers. Number and characteristics of basic emotions remained under discussion for quite some time [3]. However, in recent years it was generally agreed that anger, fear, surprise, sadness, disgust, contempt and happiness fulfil the criteria of basic emotions [4].

Emotions of opposite valence have been initially viewed as mutually exclusive in a similar fashion as “hot and cold” or “tall and short” [5–7], later studies have questioned this assumption. Pleasure and displeasure have been reported to be reciprocally related, yet not mutually exclusive concepts [8]. Concurring emotions of opposite valence can be induced while watching bittersweet video clips [9, 10]. Co-occurrence of some emotions such as happiness and sadness is thought to be rare [11], whereas a mixed state of other emotions such as amusement and disgust is fairly common [12, 13]. A recent study comprising several thousand participants, who recorded their emotions at multiple time points throughout the day, was able to demonstrate that people experience emotions most of the time [14]. On frequent occasions participants reported to experience more than one emotion at the same time.

Emotion measurements remain disputed due to validity issues of self-report [15]. It is argued that instead of reporting their own experience of emotion, participants might instead report the affective quality of the stimulus (e.g. pleasant or unpleasant) without actually being emotionally effected by it [16]. However, on viewing emotionally charged films, change of emotions was also accompanied by a change in facial expressions and other physiological responses (e.g. electrocardiogram, blood volume, skin conductance) [17]. Likewise, emotional states can be induced using pictures [18, 19], text passages [20], or videos [21, 22]. Some evidence exists that visual stimuli are more effective elicitors of discrete emotions than verbal stimuli or imaginative techniques [19]. Until now, however, no methods are established to quantify mood induction in an objective fashion and to compare the efficacy of different stimuli to induce emotions.

Several years ago analysis of facial emotion expression was standardized using the Facial Action Coding System (FACS) [23]. FACS attributes a change of activity in facial muscles or groups of muscles (‘action units’) to emotions. Manual analysis of facial expressions, however, is time consuming. In recent years automated analysis of facial expressions has gained importance and a good concordance of automated and manual analysis was shown [24].

Automated analysis of facial expressions has been used in a variety of settings. It has been shown that analysis of facial expressions gets harder when participants are asked to react to specific scenarios rather than just imitate stereotypical portrayals of facial expressions from the photographs [25]. Although a variety of techniques using pictures, music, or verbal material is known to induce subjective mood changes [19, 26–30], it remains unclear to what extent subjectively assessed mood induction corresponds to analysis of facial expressions. Furthermore, emotional expressivity is influenced by a variety of physiological characteristics such as the amount of sleep [31] and psychological factors such as self-esteem [32], which tend to change as we age [33, 34], making analysis of older faces even more challenging.

Socioemotional Selectivity Theory posits a change of emotional expressivity with aging [35–38]. It hypothesizes a greater salience of emotional information and an increased focus on emotion regulation with increasing age [38]. Accordingly, old persons were reported to regulate emotion more effectively [39] and to suppress negative emotions in daily life more successfully [36]. Moreover, attention in older persons seems to be shifted from negative to positive emotions with aging [40, 41]. However, these hypotheses and conclusions rely on subjective assessment of emotions and thus may be distorted by age-specific social desirability response bias [42] and suggestibility [43].

As inseparable and common as emotions are in daily life of both younger and older persons it remains elusive how to reliably appraise them. The goal of the present study was twofold. Firstly, it measured the success of mood induction procedures for happiness, anger, sadness, and disgust and compared it between younger and older persons. Secondly, it aimed to find out how the information of subjective mood corresponds to analysis of mood using automated analysis of facial expressions.

## Methods

The present study was designed as an experimental study and aimed to assess the influence of emotion induction on subjective and objective emotional response. All participants were briefed about the study design and signed a written consent. Experimental sessions took place at the Division of Mental Health and Old Age Psychiatry of Ulm University. The study received approval of the ethics committee of Ulm University (434/18) and was done in accordance with the ethical standards of the Ulm University and the guidelines outlined in the declaration of Helsinki [44].

### Participants

The study sample consisted of two groups—young adults (YA) and older adults (OA). Both YA and OA groups consisted of volunteers recruited by local advertising to take part in a study on emotional response to video sequences. Central nervous system disorders, including affective disorders, intellectual disability and addiction were ruled out by taking medical history. Patients with a *Mini Mental State Examination* [45] score below 27 points, digit span [46] bellow 6 and *Brief Patient Health Questionnaire 9* [47] above 9 were excluded from the study. The final study sample consisted of 29 YA (16 females, age 24.41 ± 2.26 (mean (M) ± Standard Deviation (SD)) and 28 OC (15 females, age 69.18 ± 7.41). Mean years of schooling comprised 14.69 ± 2.19 years for the YA and 14.25 ± 3.37 for the OA group.

### Assessments

#### Actual Mood State (AMS)

Subjective self-evaluation of mood state was assessed using a 7-point Likert scale ranging from 1 “not at all” to 7 “very much” for four basic emotions (happiness, sadness, anger, disgust) and general arousal. The participants were asked to evaluate their current emotional state for each emotion and arousal before and after each emotion induction video. We chose a single-item global Likert Scale as it was shown to have greater utility than visual analogue scales in other research areas relevant concerning elderly populations [48].

#### Berkeley Expressivity Questionnaire; BEQ [49]

Using 16 items and a 7-point Likert scale BEQ assesses three aspects of emotional expressivity—negative expressivity, positive expressivity and impulse strength.

#### Short Suggestibility Scale; SSS [50]

SSS is a short form of the Multidimensional Iowa Suggestibility Scale and assess one’s susceptibility to accept and internalise external influences. The scale consists of 21 items that are divided into following categories: consumer suggestibility, persuadability, sensation contagion, physiological reactivity and peer conformity.

### Film sequences used for emotion induction

All movie sequences were selected from the material previously demonstrated to elicit basic emotions in a sample of young college students [51]. The length of each video was between 105 and 165 seconds.

#### Target emotion happiness; *film—When Harry met Sally*

This film sequence shows a dialogue between two friends (Harry and Sally) in a restaurant. While eating they talk about whether men are able to recognize when women simulate having an orgasm. To challenge Harry, who is convinced he would be able to tell a difference, Sally unexpectedly starts to moan and move like having an orgasm. At the end of the scene another woman visiting the restaurant misinterprets the situation and asks the waiter to be served the same dish as Sally.

#### Target emotion sadness; film—*Dangerous Minds*

A young woman teaches a class of teenagers when she is asked by a female clerk to step outside. In the hall the clerk tells the teacher that one of the students was killed. After returning to the classroom, the teacher shares the sad news with the students, who all seem shocked and one girl starts crying.

#### Target emotion anger; film—*Schindler’s List*

The excerpt shows the commander of a concentration camp overlooking the working inmates from a balcony. The camera switches back and forth between the commander and the camp while he aims with his rifle at persons in the camp and shoots two of the prisoners. Subsequently he stretches his shoulder muscles and returns to the bedroom where a young woman lies in his bed.

#### Target emotion disgust; film—*Trainspotting*

The scene begins with a young male entering a filthy stall in a public bathroom. After initially feeling repulsed, he decides to relieve himself, but unfortunately loses one of his possessions in the toilet bowl. Determined to retrieve it he reaches his hand and later his arm down the toilet. The scene unfolds in a bizarre fashion showing how the protagonist dives into the toilet bowl and then into the sewage system.

### Distraction task

A distraction task was a simple counting task where simple objects (e.g. chair, ball, lightbulb) were presented on a screen. Participant was instructed to count the objects he sees on the screen, say the result and press a button to continue to a next counting task. The distraction task was timed at 30s and independent of participants working speed. The purpose of the task was to give participants time to return to the neutral emotional state after emotion induction.

### Procedure

After signing an informed consent participants were asked a few questions concerning their demographic information and medical history, which was followed by the MMSE, assessment of digit span and filling in SSS, BEQ and PHQ-9. After it was confirmed that participants fulfil inclusion criteria, they proceeded to an experimental part of the study, which took place on a computer. Script for the experimental paradigm was created using *E-Prime*^*®*^ software. It consisted of four analogue cycles each including two instances of AMS before and after the video, emotion induction video sequence, and a distractor task. The cycle was repeated once for every emotion with a corresponding video in a randomised order. After each video sequence participants were asked if it was known to them. During the emotion induction session participant was seated in front of a camera placed of the top of the monitor, which was analysing facial expressions using FaceReader^™^ (Version 7.0, Noldus Information Technology b.v.) software. Data from the experimental script and the camera was transmitted to a second computer behind a screen and synchronised using *Observer XT*. For data analysis we used the mean of the first 200 measurements (10 hz sampling rate) of the films for baseline. The induction was measured using the average of the remaining measurements taken while participants watched the film sequences. The total session length was about approximately 30 min.

### Statistical analyses

Statistical analysis was carried out using SPSS software (SPSS 25.0 for Windows, Armonk, NY, 2017). The normality of distribution was assessed by Shapiro-Wilk’s test and additional visual inspection of the histograms. Data are reported as mean ± standard deviation.

A three-way mixed ANOVA with two within-subject factors (emotion: happiness, sadness, anger disgust; time: pre-induction, post-induction) and one between-subject factor (group: YA and OA) was used to analyse the group differences. We also performed a two-way mixed ANOVA with one within-subject factor (time: pre-induction, post-induction) and one between-subject factor (group: YA and OA) for arousal. There was a homogeneity of variance, as assessed by Levene’s test for equality of variances (p > .05) for all comparisons. In cases, where the assumption of sphericity was violated, a Greenhouse-Geisser correction was applied when analysing the results. Differences of estimated marginal means are reported with 95% confidence intervals. Adjustment for multiple comparisons was performed by Sidak correction.

A backward linear regression was used to predict the variance in the subjective emotions and facial expressions after the emotion induction.

## Results

**Fig 1:**
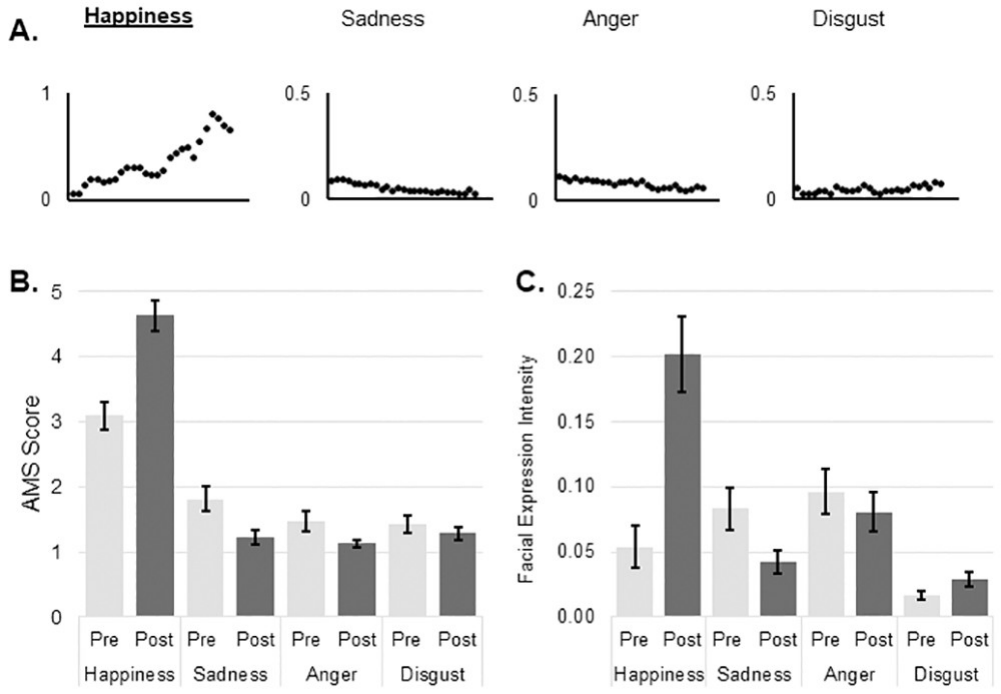
Happiness induction. A: Change in mean values of emotions during the course of video as measured by automated facial expression analysis; B: Mean and SE of subjective mood evaluation as measured by AMS; C: Mean and SE of objective mood evaluation as measured by automated facial expression analysis. AMS: Actual Mood State; SE: Standard Error.

**Fig 2:**
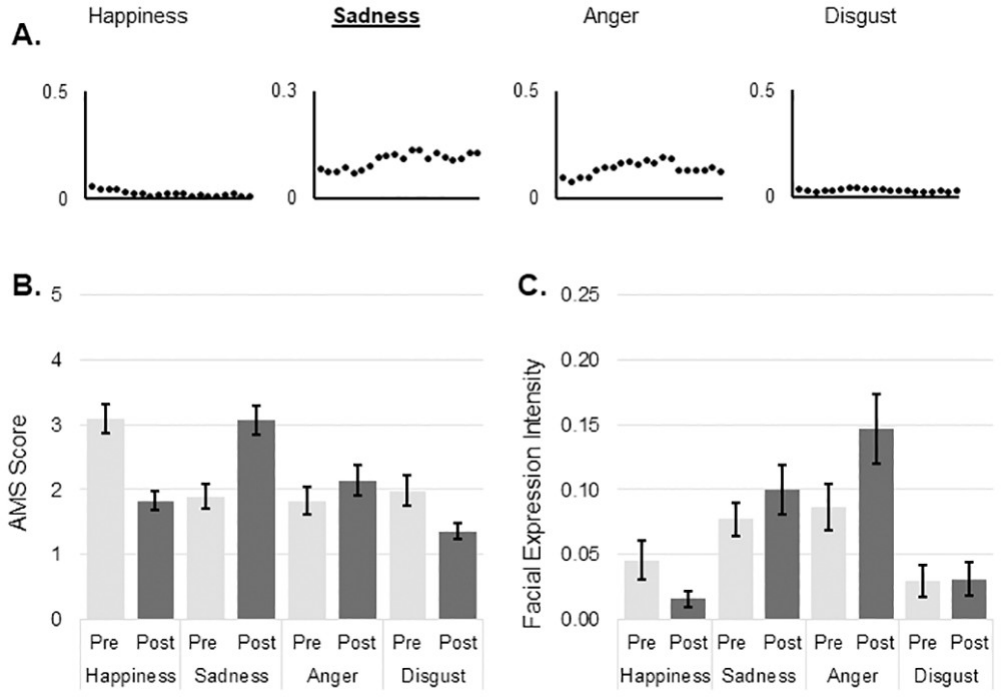
Sadness induction. A: Change in mean values of emotions during the course of video as measured by automated facial expression analysis; B: Mean and SE of subjective mood evaluation as measured by AMS; C: Mean and SE of objective mood evaluation as measured by automated facial expression analysis. AMS: Actual Mood State; SE: Standard Error.

**Fig 3:**
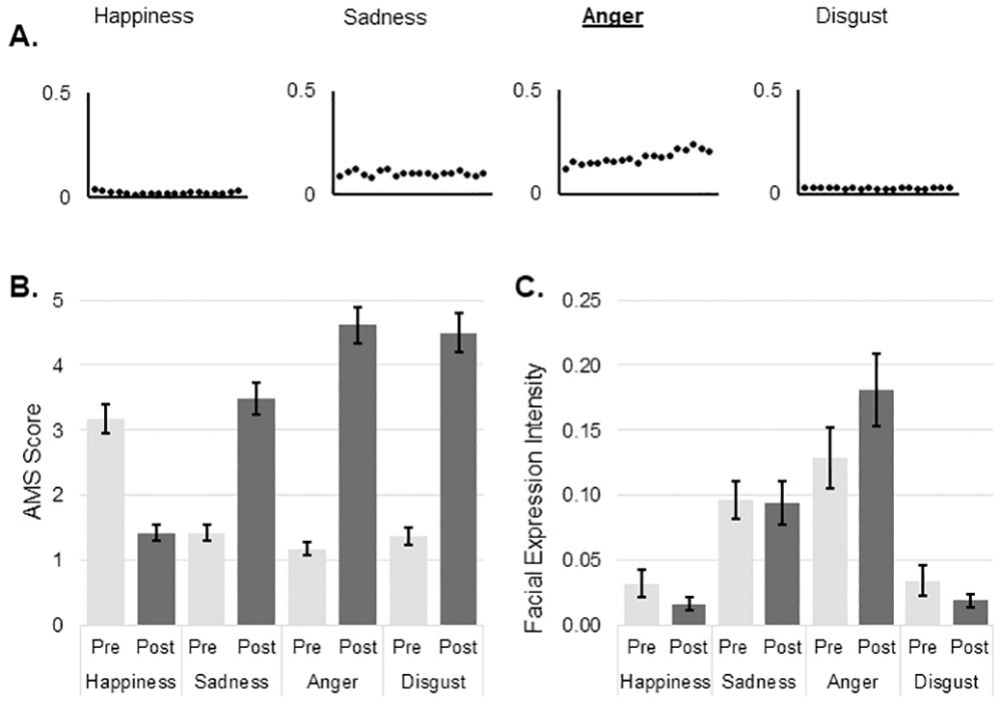
Anger induction. A: Change in mean values of emotions during the course of video as measured by automated facial expression analysis; B: Mean and SE of subjective mood evaluation as measured by AMS; C: Mean and SE of objective mood evaluation as measured by automated facial expression analysis. AMS: Actual Mood State; SE: Standard Error.

**Fig 4:**
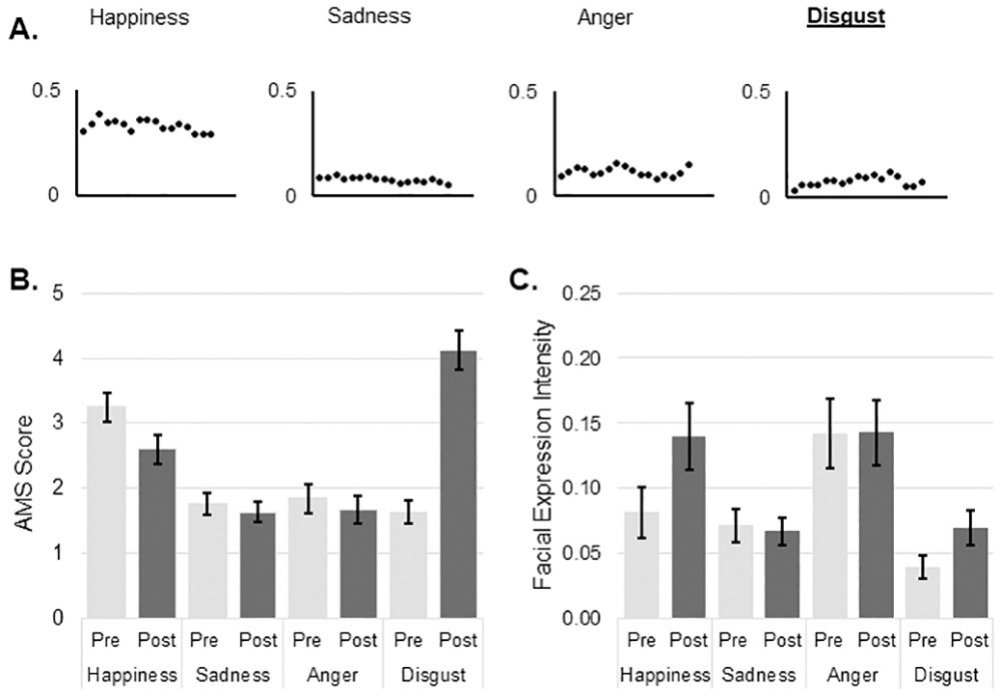
Disgust induction. A: Change in mean values of emotions during the course of video as measured by automated facial expression analysis; B: Mean and SE of subjective mood evaluation as measured by AMS; C: Mean and SE of objective mood evaluation as measured by automated facial expression analysis. AMS: Actual Mood State; SE: Standard Error.

There were no differences in the expressivity measure as assessed by BEQ (YA 63.5 ± 13.8, OA 58.3 ± 14.3, t_(55)_ = 1.387, p = .171; mean ± standard deviation), however there was a significant difference between in suggestibility measure as assessed by SSS (YA 45.5 ± 6.8, OA 37.7 ± 8.2, t_(54)_ = 3.876, p < .001; mean ± standard deviation). The distributions of mean facial expression values over the duration of each movie for happiness, sadness, anger, disgust and arousal are shown in Figs 1A–4A. The values for each emotion range between zero (emotion not expressed) and 1 (emotion expressed to maximum intensity). Means and standard deviations for subjective emotion assessment (AMS) and facial expression intensity are presented in Tables 1 and 2 respectively.

**Table 1:** Mean ± standard deviation of subjective emotions and arousal evaluation as measured by Actual Mood Scale before and after emotion induction films.

	Happiness		Sadness		Anger		Disgust		Arousal	
	Pre	Post	Pre	Post	Pre	Post	Pre	Post	Pre	Post
**Film Happy**										
All	3.09 ± 1.57	4.63 ± 1.74	1.81 ± 1.41	1.23 ± .80	1.47 ± 1.15	1.12 ± 0.43	1.42 ± .98	1.29 ± .73	2.21 ± 1.13	2.61 ± 1.22
YA	3.52 ± 1.60	4.86 ± 1.22	1.76 ± 1.22	1.17 ± .60	1.52 ± 1.02	1.03 ± 0.19	1.38 ± .78	1.10 ± .31	2.48 ± 1.21	3.07 ± 1.16
OA	2.64 ± 1.45	4.39 ± 2.15	1.86 ± 1.60	1.29 ± .98	1.43 ± 1.29	1.21 ± .57	1.48 ± 1.19	1.48 ± .98	1.93 ± .98	2.14 ± 1.11
**Film Sadness**										
All	3.09 ± 1.61	1.82 ± 1.14	1.89 ± 1.51	3.07 ± 1.72	1.82 ± 1.59	2.14 ± 1.70	1.98 ± 1.75	1.35 ± .92	2.28 ± 1.25	2.68 ± 1.21
YA	3.66 ± 1.23	2.21 ± 1.21	1.79 ± 1.08	2.72 ± 1.49	1.59 ± 1.18	1.62 ± .98	1.72 ± 1.22	1.14 ± .35	2.45 ± 1.21	2.76 ± 1.06
OA	2.48 ± 1.76	1.44 ± .93	2.00 ± 1.87	3.43 ± 1.89	2.07 ± 1.92	2.68 ± 2.09	2.25 ± 2.15	1.57 ± 1.23	2.11 ± 1.29	2.61 ± 1.37
**Film Anger**										
All	3.18 ± 1.68	1.42 ± .93	1.42 ± 1.00	3.49 ± 1.82	1.18 ± .76	4.61 ± 2.10	1.36 ± 1.00	4.49 ± 2.27	2.16 ± 1.27	3.25 ± 1.18
YA	3.79 ± 1.61	1.66 ± 1.01	1.41 ± .87	2.93 ± 1.56	1.07 ± .26	3.97 ± 1.90	1.31 ± .76	3.62 ± 1.97	2.45 ± 1.24	3.21 ± 1.01
OA	2.54 ± 1.53	1.18 ± .77	1.43 ± 1.14	4.07 ± 1.92	1.29 ± 1.05	5.29 ± 2.12	1.41 ± 1.22	5.33 ± 2.25	1.86 ± 1.24	3.29 ± 1.36
**Film Disgust**										
All	3.25 ± 1.70	2.60 ± 1.73	1.75 ± 1.21	1.63 ± 1.16	1.84 ± 1.68	1.67 ± 1.54	1.63 ± 1.40	4.12 ± 2.28	2.21 ± 1.11	2.81 ± 1.25
YA	3.76 ± 1.46	3.14 ± .64	1.79 ± .98	1.45 ± .87	1.45 ± .95	1.14 ± .35	1.41 ± .98	3.76 ± 2.08	2.34 ± 1.11	2.93 ± 1.19
OA	2.70 ± 1.79	2.07 ± 1.69	1.71 ± 1.44	1.82 ± 1.39	2.25 ± 2.14	2.21 ± 2.04	1.86 ± 1.72	4.50 ± 2.44	2.07 ± 1.12	2.68 ± 1.31

OA = older adults, YA = young adults; target emotions for each video in grey.

**Table 2:** Mean ± standard deviation of facial expression values before and after emotion induction films.

	Happiness		Sadness		Anger		Disgust		Arousal	
	Pre	Post	Pre	Post	Pre	Post	Pre	Post	Pre	Post
**Film Happy**										
All	.05 ± .12	.20 ± .22	.08 ± .12	.05 ± .07	.10 ± .13	.09 ± .12	.04 ± .12	.07 ± .12	.14 ± .14	.13 ± .11
YA	.06 ± .14	.20 ± .20	.09 ± .14	.05 ± .06	.07 ± .10	.05 ± .07	.03 ± .09	.04 ± .08	.10 ± .12	.11 ± .10
OA	.05 ± .10	.20 ± .24	.08 ± .10	.04 ± .07	.14 ± .15	.13 ± .15	.05 ± .14	.10 ± .16	.18 ± .15	.15 ± .12
**Film Sadness**										
All	.05 ± .12	.02 ± .04	.08 ± .10	.10 ±.14	.09 ± .13	.14 ± .20	.03 ± .10	.03 ± .09	.12 ± .15	.10 ± .10
YA	.06 ± .15	.02 ± .06	.08 ± .10	.12 ± .16	.04 ± .08	.07 ± .12	.00 ± .01	.00 ± .01	.07 ± .10	.07 ± .06
OA	.04 ± .08	.01 ± .03	.07 ± .09	.08 ± .12	.14 ± .16	.23 ± .23	.06 ± .13	.06 ± .13	.17 ± .18	.14 ± .11
**Film Anger**										
All	.03 ± .08	.02 ± .04	.10 ± .11	.09 ± .13	.13 ± .18	.18 ± .21	.03 ± .09	.02 ± .03	.17 ± .17	.11 ± .10
YA	.04 ± .09	.02 ± .04	.07 ± .09	.08 ± .11	.11 ± .19	.18 ± .24	.01 ± .01	.01 ± .01	.12 ± .15	.09 ± .09
OA	.03 ± .07	.02 ± .03	.12 ± .13	.11 ± .14	.15 ± .17	.18 ± .18	.06 ± .12	.03 ± .04	.22 ± .17	.13 ± .11
**Film Disgust**										
All	.35 ± .15	.32 ± .12	.07 ± .09	.07 ± .08	.14 ± .19	.15 ± .19	.04 ± .06	.07 ± .10	.19 ± .16	.16 ± .11
YA	.35 ± .13	.34 ± .10	.07 ± .09	.06 ± .08	.11 ± .18	.11 ± .16	.03 ± .06	.06 ± .07	.17 ± .10	.16 ± .10
OA	.35 ± .18	.29 ± .14	.07 ± .10	.07 ± .07	.18 ± .20	.19 ± .21	.05 ± .06	.09 ± .13	.23 ± .19	.17 ± .12

OA = older adults, YA = young adults; target emotions for each video in grey.

### Induction of happiness (film: *When Harry met Sally*)

#### Subjective evaluation

There was no statistically significant three-way interaction between subjective emotion estimation, time and group. There was a statistically significant two-way interaction between emotion and time of assessment, F _(1.726, 93.204)_ = 22.511, p < .001, partial η^2^ = .294. There was a significant increase in subjective happiness after emotion induction procedure with a mean difference of 1.469 (p < .001; 95% CI, 0.93 to 2.01) AMS points on happiness scale. Subjective sadness decreased by a mean of 0.589 (p = .004; 95% CI, 0.19 to 0.99) AMS points on sadness scale and subjective anger decreased by a mean of 0.352 (p = .017; 95% CI, 0.07 to 0.64) AMS points on anger scale (Fig 1B).

There was no statistically significant two-way interaction between group and time of assessment on subjective arousal estimates, but there was a significant main effect of time of assessment (F _(1, 55)_ = 8.403, p = .005, partial η^2^ = .133). Subjective arousal was significantly higher after emotion induction (mean difference 0.40, 95% CI 0.12 to 0.68). There was also a significant main effect of group (F _(1, 55)_ = 7.911, p = .007, partial η^2^ = .126) on arousal. YA group rated their arousal higher than OA group (mean difference 0.74, 95% CI 0.21 to 1.27).

#### Facial expression

There was no statistically significant three-way interaction between emotion, time and group. A two-way interaction between emotion and time of assessment was significant, F _(1.809, 95.882)_ = 22.469, p < .001, partial η^2^ = .298. Intensity of facial happiness increased significantly after happiness induction (a mean difference of 0.151, p < .001, 95% CI 0.10 to 0.21). Likewise, there was a significant decrease in expressed facial sadness (a mean difference of 0.041, p = .002, 95% CI, 0.02 to 0.07) and slight but statistically significant increase in facial disgust (a mean difference of 0.013, p = .020, 95% CI, 0.002 to .02) as measured by facial expression intensity (Fig 1C).

There was no statistically significant two-way interaction between group and time of assessment on expressed facial arousal. A main effect of group was statistically significant (F _(1, 53)_ = 8.042, p = .006, partial η^2^ = .132) with OA showing higher level of facial arousal than YA (mean difference 0.08, 95% CI 0.02 to 0.14).

### Induction of sadness (film: *Dangerous Minds*)

#### Subjective evaluation

There was no statistically significant three-way interaction between group, emotion and time of assessment on AMS scores after sadness induction. There was a significant two-way interaction between emotion and time, F _(2.256, 121.814)_ = 29.072, p < .001, partial η^2^ = .350. Sadness increased significantly after viewing the video clip with a mean difference of 1.188 (p < .001; 95%CI 0.75 to 1.62). Simultaneously subjective scores of happiness decreased with a mean difference of 1.243 (p < 0.001; 95% CI 1.63 to 0.86) and scores for disgust decreased by a mean difference of 0.645 (p = .004; 95%CI 1.08 to 0.21) (Fig 2B). There was also a significant interaction between emotion and group on subjective emotion evaluations, F _(1.767, 95.423)_ = 8.798, p = .001, partial η^2^ = .140. Subjective happiness was higher in YA compared to OA by a mean difference of 0.968 (95% CI .38 to 1.56) AMS points.

There was no statistically significant two-way interaction between group and time of assessment on subjective arousal estimates, but there was a significant main effect of time of assessment (F _(1, 55)_ = 13.828, p < .001, partial η^2^ = .201). Subjective arousal was significantly higher after emotion induction (mean difference 0.41, 95% CI 0.19 to 0.62).

#### Facial expression

There was no statistically significant three-way interaction between group, emotion and time of assessment on expressed facial emotions. There was a significant two-way interaction between emotion and time of assessment, F _(1.691, 77.803)_ = 8.864, p = .001, partial η^2^ = .162. Expressed facial happiness decreased significantly by a mean difference of 0.031 (p = .016, 95% CI 0.01 to 0.06) and expressed facial anger increased significantly by a mean of 0.061 (p = .002, 95% CI 0.02 to 0.10) after sadness induction (Fig 2C). There was also a significant interaction between emotion and group, F _(2.422, 111.422)_ = 4.865, p = .006, partial η^2^ = .096. Expressed facial anger (a mean difference of 0.124, p = .005, 95% CI 0.04 to 0.21) and disgust (a mean difference of .054, p = .048, 95% CI 0.001 to 0.11) were significantly higher in older adults.

There was no statistically significant two-way interaction between group and time of assessment on expressed facial arousal. A main effect of group was statistically significant (F _(1, 51)_ = 8.037, p = .007, partial η^2^ = .136) with OA showing higher level of facial arousal than YA (mean difference 0.08, 95% CI 0.02 to 0.14).

### Induction of anger (film: *Schindler’s List*)

#### Subjective evaluation

There was no three-way interaction between group, emotion and time of assessment after anger induction procedure. There was a two-way interaction between emotion and time, F _(2.396, 129.402)_ = 99.906, p < .001, partial η^2^ = .649. After watching the video participants rated their anger as significantly higher (a mean difference of 3.448, p < .001 (95% CI 2.89 to 4.00). At the same time participants experiences a significant subjective increase in sadness (a mean difference of 2.018, p < .001, 95% CI 1.55 to 2.49) and disgust (a mean difference of 3.118, p < .001, 95% CI 2.50 to 3.74). Subjective happiness scores dropped by a mean difference of 1.717 (p < .001, 95% CI 1.32 to 2.12; Fig 3B). There was also a significant two-way interaction between emotion and group, F _(2.209, 119.264)_ = 10.293, p < .001, partial η^2^ = .160. Older adults reported more subjective anger (a mean difference of 0.78, p = .013, 95% CI 0.17 to 1.39) and disgust (a mean difference of 0.91, p = .006, 95% CI 0.27 to 1.54). At the same time OA reported less happiness (a mean difference of 0.891, p = .002, 95%CI 0.33 to 1.45).

There was no significant two-way interaction between group and time of assessment on subjective arousal estimates. Main effect of time of assessment was significant, (F _(1, 55)_ = 47.950, p < .001, partial η^2^ = .466). Subjective arousal was significantly higher after emotion induction (mean difference 1.09, 95% CI 0.78 to 1.41).

#### Facial expression

There was no three-way interaction between group, emotion and time of assessment on facial expression intensity after anger induction procedure. There was a significant interaction between emotion and time, F _(2.361, 120.425)_ = 6.206, p = .002, partial η^2^ = .108. Expressed facial anger was significantly higher after emotion induction than before (a mean difference of 0.051, p = .008, 95% CI 0.01 to 0.09; Fig 3C).

There was no statistically significant two-way interaction between group and time of assessment on expressed facial arousal. A main effect of time of assessment was statistically significant (F _(1, 51)_ = 10.899, p = .002, partial η^2^ = .171) with pre-induction values being slightly higher than post induction (mean difference 0.06, 95% CI 0.02 to 0.09). There was also a statistically significant main effect of group (F _(1, 53)_ = 4.450, p = .040, partial η^2^ = .077), with older adults showing higher facial arousal than younger adults (mean difference 0.07, 95% CI 0.003 to 0.128).

### Induction of disgust (film: *Trainspotting*)

#### Subjective evaluation

There was no significant three-way interaction between emotion, group and time of assessment on subjective emotion ratings after induction of disgust. An interaction between emotion and time was significant, F _(2.175, 117.431)_ = 49.013, p < .001, partial η^2^ = .476. After watching the video clip subjective disgust increased by a mean difference of 2.49 (p < .001, 95%CI 1.91 to 3.07). Simultaneously there was a significant decrease in subjective happiness (a mean difference of 0.625, p = .001, 95% CI 0.26 to 0.99; Fig 4B). There was also a significant interaction between emotion and group, F _(1.839, 99.283)_ = 6.878, p < .001, partial η^2^ = .113. As a result of disgust induction procedure YA reported significantly more happiness (a mean difference of 1.059, p = .011, 95% CI 0.26 to 1.86); whereas OA reported significantly more anger (a mean difference of 0.985, p = .012, 95% CI 0.23 to 1.74).

There was no statistically significant two-way interaction between group and time of assessment on subjective arousal estimates, but there was a significant main effect of time of assessment (F _(1, 55)_ = 28.096, p < .001, partial η^2^ = .338). Subjective arousal was significantly higher after emotion induction (mean difference 0.58, 95% CI 0.37 to 0.82).

#### Facial expression

There was no significant three-way interaction between emotion, group and time of assessment on expressed facial disgust. There was a significant two-way interaction between emotion and time of assessment, F _(2.165, 108.225)_ = 3.292, p = .037, partial η^2^ = .062. Subjective ratings of disgust increased by a mean difference of 0.036 (p = .001, 95% CI 0.02 to 0.06). Similarly facial expressions of happiness also increased by a mean difference of 0.062 (p = .011, 95% CI 0.02 to 0.11; Fig 4C) indicating that participants were not only disgusted, but also amused by the video.

There was no statistically significant two-way interaction between group and time of assessment as well as no significant main effects on expressed facial arousal after disgust induction procedure.

### Explanatory factors for facial expressions

A linear regression analysis using a stepwise backward elimination was performed to identify the contributing factors to facial expressions of target emotions as well as subjective value of target emotions after a corresponding emotion induction film sequence. Pre-induction values of corresponding subjective emotion (as measured by AMS), pre-induction values of corresponding facial expression (as measured by automated facial expression analysis), BEQ score, and SSS score were entered into the model to predict post-induction subjective emotion intensity as well as post-induction facial expression values. The model for facial expression values predicted at least 50% of variance in disgust, anger and sadness as well as 23% of variance in facial happiness (Table 3). The pre-induction values of corresponding emotions were the strongest predictors. The models for post-induction values of subjective emotions could predict 18% variance in subjective disgust and 27% variance in subjective sadness, with subjective pre-induction values or corresponding emotions being the strongest predictors (Table 4). Only 5% of variance in subjective happiness and only 9% of variance in subjective anger could be predicted using the model, with BEQ and SSS values explaining some of the variance.

**Table 3:** Regression model for facial expressions after corresponding emotion induction.

	R	Adj. R^2^	Regression coefficient B	Standard error	Standardized coefficient Beta	t	p
**Happiness**							
Model*	.506	.228					
Facial Happiness			.911	.220	.499	4.148	< .001
AMS Happiness			-.029	.017	-.208	-1.729	.090
**Sadness**							
Model*	.764	.576					
Facial Sadness			1.131	.131	.764	8.625	< .001
**Anger**							
Model*	.801	.629					
SSS			.005	.002	.183	2.230	.030
Facial Anger			.920	.097	.779	9.476	< .001
**Disgust**							
Model*	.722	.503					
BEQ			.001	.001	.170	1.760	.084
Facial Disgust			1.164	.159	.707	7.301	< .001

*Model including BEQ, SSS, pre-induction facial expression value of target emotion, pre-onduction AMS value of target emotion

AMS = Actual Mood State, BEQ = Berkley Expressvity Questionnaire, SSS = Short Suggestibility Scale.

**Table 4:** Regression model for subjective emotion after corresponding emotion induction.

	R	Adj. R^2^	Regression coefficient B	Standard error	Standardized coefficient Beta	t	p
**Happiness**							
Model*	.260	.050					
SSS			.054	.027	.260	1.977	.053
**Sadness**							
Model*	.548	.274					
Facial Sadness			-3.705	2.086	-.204	-1.776	.082
AMS Sadness			.579	.132	.505	4.397	< .001
**Anger**							
Model*	.350	.089					
BEQ			.057	.021	.383	2.650	.011
SSS			-.063	.036	-.251	-1.737	.088
**Disgust**							
Model*	.458	.178					
BEQ			.040	.020	.252	2.013	.049
AMS Disgust			.634	.220	.361	2.884	.006

*Model including BEQ, SSS, pre-induction facial expression value of target emotion, pre-onduction AMS value of target emotion

AMS = Actual Mood State, BEQ = Berkley Expressvity Questionnaire, SSS = Short Suggestibility Scale.

## Discussion

The goal of the present study was to assess mood induction procedures for happiness, anger, sadness, disgust in younger and older persons. The analysis shows that mood induction is successful for all emotions targeted (happiness, anger, sadness, and disgust). Mood induction was successful and multivariate analysis did not reveal age differences regarding the success of mood induction.

Few studies have assessed the age-dependency of mood induction. Some studies found mood induction for fear, anger, sadness and disgust to be even more intense in older than in younger subjects [52, 53]. On the other hand older adults have been reported to have a more effective emotion regulation, which enables them to recover quicker after confrontation with emotional and in particular negative stimuli [54]. Such rapid emotion regulation might have an impact on self-appraisal of mood after emotion induction procedures. Overall, mood induction is more heterogeneous in older than in younger adults [55]. Thus, the generalizability of findings on the age-specificity of mood induction is limited. Due to the lack of a gold standard, conclusions need to be limited to the specific instruments used for mood induction and the instruments with which the change of mood is measured.

On viewing emotionally charged films it is not only the target emotion that changes but also other emotions. When selecting the film sequences used in the present study we picked films from established databases of film segments for mood induction that influenced the target emotion to a much higher degree than related or opposite emotions. Nevertheless even in these films a manifold of emotions changed on exposure to these films [51]. This supports the notion that even basic emotions do not respond in an itemized fashion with all other emotions remaining at pre-stimulus level. In particular this applies to emotions that are related like fear and sadness and even emotions that seem to have opposite valence such as sadness and happiness. For example, induction of anger in the present study was associated with an increase in sadness and a decrease in happiness. This is in good harmony with previous reports demonstrating the simultaneous co-occurrence of multiple basic emotions [14] and the induction of mixed emotional states [9, 10].

In addition to mood induction in different age groups, this study aimed to find out how subjective mood corresponds to analysis of mood using automated analysis of facial expressions. The present study demonstrates that the success of mood induction can be objectified with analysis of facial expressions for all the emotions targeted.

It has been previously reported that the recognisability of a particular facial emotion may change as a function of age. It was reported that older people’s faces have a weaker signal clarity [56] due to multiple changes in facial morphology [57–60]. Angry, sad and disgusted faces are best recognized when the face portraying these emotions is in its 20s and 30s [61]. Happy facial expressions, on the other hand, did not have a clear peak but recognisability of this facial expression somewhat declined between late 50s and early 60s [61]. Thus, facial expression in older persons is confounded by the particulars of aging faces. However, the latter study used static facial expressions where subjects were asked to imitate emotions without having been presented an emotional stimulus. The present study finds no effect of age on induction of any of the target emotions was found. In contrast to the above studies we recorded a baseline at onset of automated facial analysis prior to induction of emotion. While older faces at rest may show a different propensity to reveal emotions it seems that the assessment of the change of the emotion from baseline in individual faces in older subjects is as reliable as in younger faces. Only for the target emotion sad we observed a difference between young and old faces. This might have resulted from “smile wrinkles” preventing classification as sad [62] even after successful mood induction as assessed by subjective rating. General similarity between young and older adults in their response to emotion induction using film sequences has been previously reported [63].

It was observed several years ago that the success of mood induction may be influenced by mood state prior to applying the mood induction procedure [64]. This effect is only partly supported by the results of the present study. Using a regression analysis we found pre-exposure mood to be associated with post-induction success for disgust and sadness. The association between pre-exposure facial expression of mood state and post-exposure intensity of facial expression was much more clear-cut for all emotions. This observation corresponds with the facial feedback hypothesis that the emotional facial action modulates the subjective experience of emotion [65]. In contrast, association of the trait of expressivity as assessed with the Berkeley Expressivity Questionnaire and the Short Suggestibility Scale is much tighter with self-assessment of mood than with facial expressions. We interpret this as to indicate that self-assessment of mood state may overestimate an underlying inner experience of feeling because the personality determines how strong emotions are subjectively perceived. In contrast, analysis of facial expression may underestimate the inner experience of feeling the emotion because the baseline emotion determines the difference between pre- and post-exposure measurement and if for whatever reason the facial expression does not already show signs of the target emotion at baseline the post-exposure face will show less corresponding facial activity compared to the situation when the target emotion is already reflected at baseline. This possibly indicates that at onset of the film, which is used to determine baseline of facial expression, the general setting of the film scene is already suggestive of the emotion induced over the complete sequence.

## Conclusion

Regardless of age, mood induction is successful using film clips targeting these emotions. Analysis of facial expression complements self-assessment of mood and may serve as a means of objectification of mood change in both young and old persons. The concordance between self-assessment of mood change and facial expression is modulated by personality factors.

## Supporting information

S1 Dataset (SAV)
